# Sex- and age- differences in the expression of critical blood-brain barrier regulators: a physiological context

**DOI:** 10.1186/s13293-025-00751-2

**Published:** 2025-09-02

**Authors:** Xue Mi, Zi-Ling Ye, Xu-Jun Zhang, Xiao-Chun Chen, Xiao-Man Dai

**Affiliations:** 1 Public Technology Service Center, Fujian Medical University, Fuzhou, 350122 China; 2 Department of Neurology, Fujian Key Laboratory of Molecular Neurology and Institute of Neuroscience, Fujian Medical University Union Hospital, Fujian Medical University, Fuzhou, 350001 China; 3 School of Basic Medical Sciences, Fujian Medical University, Fuzhou, 350122 China

**Keywords:** Blood-brain barrier, Sex difference, Age, Brain microvessel, Glycocalyx, Pericytes

## Abstract

**Background:**

Available evidence indicates that blood-brain-barrier (BBB) dysfunction exacerbates with the advancing age and is implicated in a variety of neurological diseases and that there are significant sex differences in these diseases. However, the sex differences and age-related changes in BBB structure and function are still unclear under physiological conditions.

**Methods:**

In this study, the mRNA was extracted from the cortical tissues and brain microvessels of male and female mice aged 3 months and 10 months to detect the expression of important BBB-related genes by qPCR.

**Results:**

Under physiological conditions, compared with age-matched male counterparts, female mice reported a significantly lower mRNA expression of tight junction-related genes (cldn5 and occludin), transporters (Glut1 and D-gp), pericyte marker (Pdgfrb), microvessel marker (Cd31), basement membrane component (Col4a2), glycocalyx-related genes (Hs3st1, Extl2, and Clgalt), vascular homeostasis-related genes (Hif1a, Ddit4, and Pik3ca), and some regulatory genes (Adm, Zfpm2 and Nr3c1). A similar outcome was found in the 10-month mice when compared with the 3-month counterparts.

**Conclusion:**

This study systematically analyzes the expression characteristics of key BBB regulatory genes in different sexes and ages under physiological conditions and reveals a marked sex difference in the expression of BBB structure/function-related genes, which may persist with the advancing age. The findings may provide important theoretical insights into the pathogenesis of sex-and age-related neurological diseases.

**Supplementary Information:**

The online version contains supplementary material available at 10.1186/s13293-025-00751-2.

## Highlights

1. Female mice exhibit a significantly lower expression of BBB-related structure/function genes (e.g., tight junctions, transporters, pericytes, glycocalyx) than age-matched males under physiological conditions.
2. Aging (10 vs. 3 months) reduces the expression of BBB structure/function-related genes in mice.
3. Sex differences in the expression of BBB structure/function-related genes persist with advancing age.
4. The marked sex-specific expression differences of key BBB structural/functional genes under physiological conditions may contribute to the varying susceptibility to neurological diseases.

**Supplementary Information:**

The online version contains supplementary material available at 10.1186/s13293-025-00751-2.

## Background

As a special physiological interface between the circulatory system and the central nervous system (CNS), the blood-brain barrier (BBB) features unique dynamic regulatory properties. The main functions of the BBB are to maintain the homeostasis of the cerebral environment, dynamically regulate the transport of substances, and fence off the exogenous damage. These functions are derived from its highly specialized multicellular complex structure. The anatomical substrate of the BBB is the cerebral microvascular endothelium, which, together with perivascular elements such as astrocyte end-feet, pericytes, basement membranes, and neurons, constitutes the neurovascular unit (NVU), a construct crucial for the stability of the CNS [1, 2]. This structural and functional complexity, however, makes the BBB particularly prone to dysfunction. The malfunction of any component unit may cause abnormal barrier permeability, triggering a neuroinflammatory response and ultimately a cascade of pathological reactions in neurodegenerative diseases [3]. This pathophysiological mechanism has been fully confirmed in neurological diseases such as Alzheimer’s disease (AD) [4], Parkinson’s disease [5, 6], Huntington’s disease [7], depression [8], schizophrenia [9], stroke [10] and multiple sclerosis [11]. Therefore, preserving BBB structure and function, including restoring BBB tight junctions and transporters, reducing neuroinflammation, and repairing blood vessels, has become a treatment strategy for neurological diseases [12].

BBB function is precisely regulated by multidimensional factors, such as BBB structural constituents, genes, age, and sex. The integrity of BBB structure is closely governed by tight junctions, BBB transporters, pericytes, endothelial glycocalyx, etc. As an important structural and functional basis of the BBB, tight junctions are composed of a variety of proteins, including the claudin family, occludin, and junctional adhesion molecules. These proteins form a continuous ribbon structure between the cell membranes of adjacent cells, tightly connecting the cells together like a zipper and strictly restricting the entry of various substances in the blood into the brain tissue [13]. Damage to the tight junction structure will increase the BBB permeability, allowing the entry of harmful substances, bacteria, viruses, etc. in the blood. Meanwhile, under physiological conditions, the BBB prevents many macromolecules from entering the brain, but special transporters on the BBB can facilitate the exchange of some small molecules, such as glucose, amino acids, ions, and small molecule drugs, across the barrier. Available evidence shows that the spatiotemporal expression changes of BBB transporters can directly affect the energy metabolism and neurotransmitter balance in the brain. For example, the reduction of Glut1 in AD can lead to cerebrovascular degeneration, reduced blood flow, and BBB rupture, which in turn aggravates AD neuron loss and neurodegenerative lesions [14].

Pericytes are found on the abluminal surface of endothelial cells of capillaries and play an essential role in the formation and function of the BBB [15, 16]. These cells play an important role in maintaining vascular stability, regulating cerebral microcirculation and blood flow [17] and controlling the BBB integrity and function [18]. Absolute pericyte coverage determines the relative vascular permeability and pericyte deficiency can increase cerebral vascular permeability [19–21]. On the other hand, brain endothelial glycocalyx is a key structural component of the BBB [22], which is a complex (carbohydrate-rich) reticular structure covering the lumen of the BBB [23]. It is mainly composed of proteoglycans, glycoproteins and glycolipids, and constitutes the first interface between blood and brain blood vessels, playing an important role in cell conduction, adhesion, transport, and morphology maintenance [24]. In the course of aging and neurodegenerative diseases, cerebral endothelial glycocalyx is highly dysregulated, which in turn causes the BBB dysfunction [25, 26].

With a normal aging process often come the changes in the vascular microenvironment, including endothelial dysfunction, increased oxidative stress, and accumulation of inflammatory factors [27, 28]. These changes can reduce cerebral blood flow, which in turn causes chronic hypoxia. The latter has been found to destroy the tight junctions of brain microvascular endothelial cells by activating the hypoxia inducible factor (HIF) pathway [29] and activate the molecular damage cascade, causing incomplete infarction of brain tissue. Studies have documented that hypoxia-induced neuroinflammation can activate microglia and aggravate oxidative stress, promoting BBB leakage [30, 31]. Chronic hypoxia and BBB destruction can set in motion a vicious cycle, accelerating the progression of neurodegenerative diseases [32].

Still, sex is another factor that is involved in the maintenance of BBB structure and functions. Current studies have documented the beneficial effects of both estrogen and androgen on the retainment of BBB integrity [33]. For example, in male mice, chronic testosterone deficiency may increase the BBB permeability to endogenous immunoglobulins and damage the integrity of tight junctions, which can be restored after testosterone supplementation [34]. In ovariectomized female rats, estrogen replacement therapy (17β-estradiol) can retain the BBB integrity [35] and enhance the linkage between the tight junctions [36]. Basic biological variables as they are, the differences in sex and age may have potential effects on the structure and function of the BBB. Yet, there is still a lack of systematic research on the involvement of sex- and age-related differences in BBB structure and function under physiological conditions.

This study attempted to systematically detect the expression changes of BBB-related genes in male and female mice of different ages under physiological conditions, providing key data for analyzing the molecular mechanism underlying the role of sex and age in the regulation of BBB function. The findings may provide an innovative theoretical basis for the understanding of the pathogenesis of neurodegenerative diseases and the promotion of relevant personalized treatments.

## Materials and methods

### Animals

This study used 3-month-old and 10-month-old C57/BL6J mice as experimental subjects, each age group consisting of an equal number of female and male mice (*n* = 6 mice/sex). The animals (6 mice per cage) were kept in a standardized environment, with water and food accessed freely. The environment was lit on a 12-hour day and night cycle (lights on at 7:00 am every day) and the humidity was controlled at 50 ± 10% and the temperature at 22 ± 1℃.

All experimental protocols involving animals were strictly reviewed by the Animal Care and Use Ethics Committee of Fujian Medical University (IACUC FJMU 2024 − 0406). The experimental operations strictly observed the European Community’s “Directive on the Care and Use of Laboratory Animals (2010/63/EU)” to ensure a full implementation of animal welfare and experimental ethics requirements.

### Cortex tissue and microvessel isolation

After the mice (all in diestrus) were perfused with 0.01 M phosphate-buffered saline (PBS), the brain tissue was quickly removed and placed in a pre-cooled PBS solution. The cortex was precisely cut, quickly frozen with liquid nitrogen, and immediately stored in a refrigerator (−80 ℃).

Microvessel isolation was performed according to a previous study [37]. Specifically, the brain tissue was separated after perfusion and the olfactory bulb was discarded. Next, meningeal vessels were removed by gently rolling on the blotting paper. Brains were minced with a razor blade on ice and then homogenized, with a loose-fit, 20 mL Dounce, in Hanks’ balanced salt solution (HBSS, Gibco) with 1% HEPES (1 M, Gibco) with twenty strokes. The homogenized tissues were centrifuged at 2,000 g for 10 min at 4 °C. Cell pellets were resuspended in 20 mL of 18% (wt/vol) dextran (Sigma) in HBSS with 1% HEPES (1 M). After homogenization, it was shaken manually for 1 min and centrifuged at 4,400 g for 15 min at 4 °C. Myelin and parenchymal cell layers were removed. Pelleted microvessels were deposited on a pre-wet 20-µm strainer, washed with ice-cold HBSS containing 1% HEPES (1 M) and 1% (wt/vol) bovine serum albumin (BSA). The filter was recovered with clean forceps and immediately immersed in the ice-cold HBSS containing 1% HEPES (1 M) and 1% (wt/vol) BSA. The vessels were detached from the filter through a gentle shake. The suspension was centrifuged at 2,000 g for 5 min at 4 °C. Ultimately, the precipitate was the enriched collection of brain microvessels for subsequent experiments.

### RNA extraction and real-time quantitative PCR (RT-qPCR)

The Rt-qPCR experiment was performed according to a previous study [38]. The total RNA was respectively extracted from cortex and brain microvessels with a TriZol reagent (Cat. No. R401-01; Vazyme, Nanjing, China). Next, the reverse transcription was performed with HiScript II Q RT SuperMix (Cat. No. R223-01; Vazyme, Nanjing, China) to synthesize cDNA templates for RT-qPCR, in strict accordance with standard operating procedures. The RT-qPCR amplification was conducted with Hieff UNICON^®^ advanced qPCR SYBR Master (Cat. No. 11185ES08, Shanghai Yasen) as the reaction system and the mouse β-actin as the internal reference gene. The relative expression of the gene was calculated by the classic 2^−ΔΔCT^ method. The RT-qPCR primer sequences used in this study are detailed in Table 1. Melting curves analysis (Figure S1) and efficiency analysis (Figure S2 and Table S1) for each primer pair were summarized in the supplementary Date.

**Table 1:** Primers of the genes investigated in the RT-qPCR analysis

gene		Primer sequences	
Adm	F	AGCTGGTTTCCATCACCCTG	
	R	TCTCATCAGCGAGTCCCGTA	
Ager	F	TGGCGAAAACGACAACCCAG	
	R	TCTCCGCTTCCTCTGACTGAT	
Angpt1	F	GTTGGTGGTTCGATGCCTGT	
	R	CATGGTGGTGGAACGTAAGGA	
B3gnt3	F	TACGGCGACATTCTCCAGTG	
	R	CACATCGTCGTCCCCATTGA	
BCRP	F	GCCTCTTGGTGAATCTCAGAAC	
	R	CTGTTGTCCGTTACATTGAATCCT	
C1galt1	F	TGTGGACAACCTGAGATGGC	
	R	GCTCCTCCGCTCATGTATCC	
Cd31	F	CTGGTACCGATCCAGGTGTG	
	R	GTTGCTGGGTCATTGGAGGT	
Cldn1	F	TTCTCTGGGATGGATCGGCT	
	R	TCCCTCGTAGATGGCCTGAG	
Cldn5	F	GCTCTCAGAGTCCGTTGACC	
	R	CTGCCCTTTCAGGTTAGCAG	
Col4a2	F	AGAGGCCAACACACTTCCAG	
	R	ATTCCTGGTGCGCCTACATC	
Cp	F	CGGATCACTACACAGGTGGC	
	R	CCATTCCACCTCTACGGCTG	
Cspg4	F	GCATCATCATTCCGGTGTGC	
	R	GGTCAACACCTGGACATCGT	
Cxcr4	F	AGCTAAGGAGCATGACGGAC	
	R	TGAAGGCCAGGATGAGAACG	
Ddit4	F	ACCTTTCAGTTGACCCTGGTG	
	R	TTGATGACTCTGAAGCCGGT	
Extl2	F	TCACCCGATCCCTGTCATCT	
	R	TCGTCTACCATCAACACCGC	
Galnt10	F	GTTTCTACTCTGCTGATGGGC	
	R	ACAATGGCCTCATGGCAAGG	
Galnt2	F	TTACTGTGGTGGACCGTTCG	
	R	GCAGTTTGGAGTTGCCTTCG	
Gfap	F	TTCGCACTCAATACGAGGCA	
	R	GTCGTTAGCTTCGTGCTTGG	
Glut1	F	ACCATCTTGGAGCTGTTCCG	
	R	GCCTTCTCGAAGATGCTCGT	
Gpc5	F	CTTAGGCTGCATGGGTCCTTC	
	R	CGCTGAGCATAGCTTTTGACTA	
Hif1α	F	AGATTTGGAGATGCTGGCTCC	
	R	CAGTGGCAGTGATGGTAGGT	
Hs3st1	F	GTCGGCTGAACCTGGACTAC	
	R	AGTTCGAGGCGTTGATCTGT	
Lama5	F	ACGTTATTGGCCGTGACTGT	
	R	GTGCGTGGTGGACAGATACA	
LAT1	F	ATGATGTGGCTCCGATTCAAGAAG	
	R	TTCCAGAATGACACGGCAATGAG	
Lrp1	F	CCACTATGGATGCCCCTAAAAC	
	R	GCAATCTCTTTCACCGTCACA	
Mmp9	F	ATGTCACTTTCCCTTCACCTTC	
	R	TGCCGTCCTTATCGTAGTCA	
NeuN	F	GTGCTGAGATTTATGGAGGCTATG	
	R	ATGGTTCCGATGCTGTAGGT	
Nr3c1	F	AAGGCGATACCAGGATTCAGA	
	R	AGCATAGCAGGTTTCCACTTG	
Occludin	F	ATGGCTGCTGCTGATGAATA	
	R	CTTGATGTGCGATAATTTGCTCTT	
Pdgfrb	F	CAGCCAGAAGTAGCGAGAAG	
	R	GCAGTATTCCGTGATGATGTAGAT	
P-gp	F	AGCCTAGCCGCTTCATCTTC	
	R	TCCGGTGAGGGTCAGGATAG	
Pik3cα	F	GACCTGTTCACTCGGTCCTG	
	R	ACACGTTCCCGCTTATAGCC	
Sdc4	F	TCTTGGCAGCTCTGATCGTG	
	R	AGTCGTAACTGCCTTCGTCC	
Socs3	F	AGAAGATTCCGCTGGTACTGA	
	R	GCTGGGTCACTTTCTCATAGG	
Timp-3	F	AGGGCCTCAATTACCGCTAC	
	R	GAGCATGTCGGTCCAGAGAC	
Tjp1	F	GAGCAAGCCTTCTGCACATC	
	R	TCGGGTTTTCCCTTTGAAGAGT	
Ttr	F	TCGTACTGGAAGACACTTGGC	
	R	GTGCTGTAGGAGTATGGGCTG	
Zfpm2	F	TTCCGGTTCCCAAGTGTGAC	
	R	TTCTGCTTGTGCGCCAGATA	
β-actin	F	TGTCCACCTTCCAGCAGATGT	
	R	AGCTCAGTAACAGTCCGCCTAG	

### Statistical analysis

Data were presented as Mean ± SEM and statistically analyzed with GraphPad Prism 9.0 software. The differences between groups were assessed by the Unpaired two-sample *t*-test. The statistical difference was designated at *p* < 0.05.

## Results

### BBB permeability and BBB transporters

**Fig. 1:**
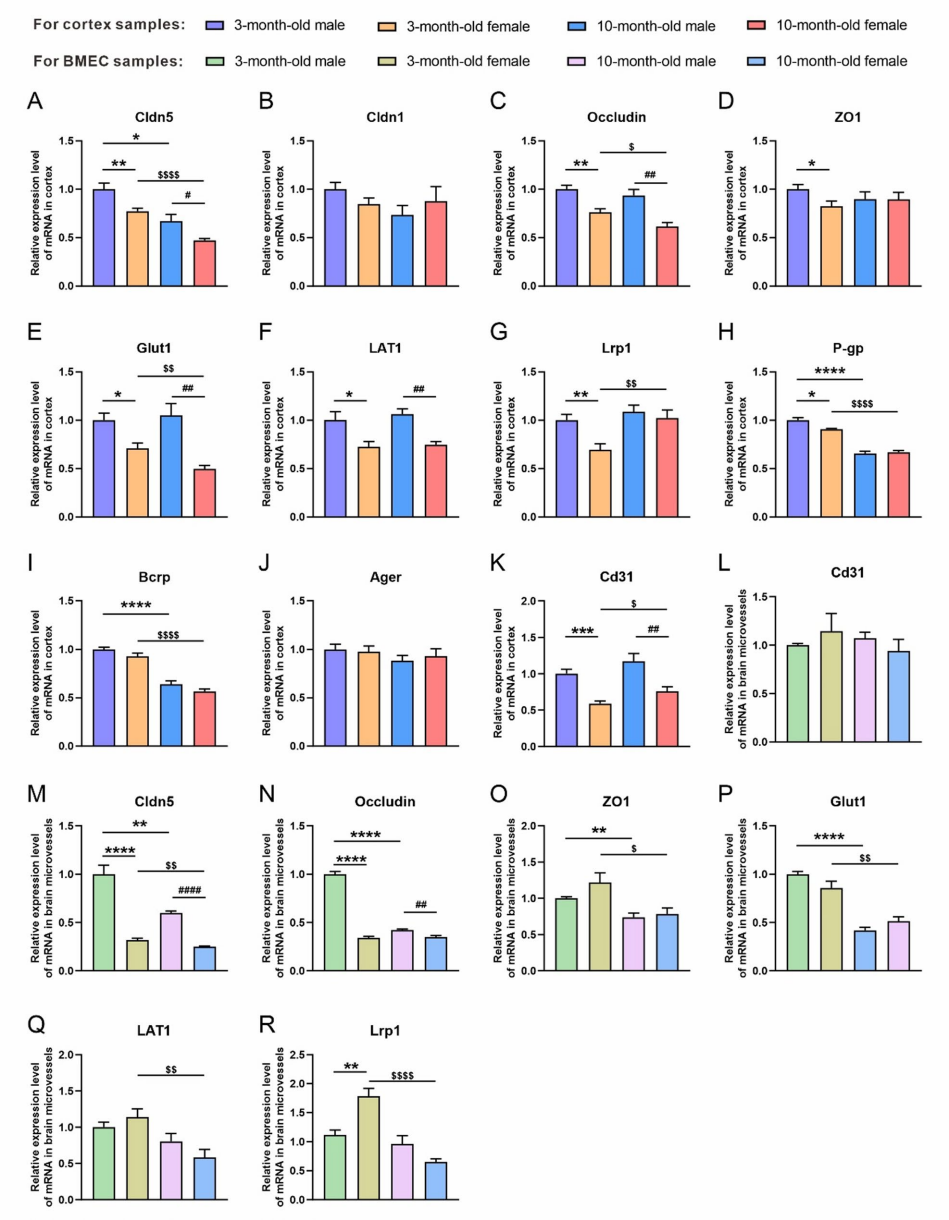
The mRNA levels of BBB tight junction- and transporter-related genes in the cortex and brain microvascular samples of male and female mice at 3 and 10 months of age. (A-K) The mRNA levels of Cldn5 (**A**), Cldn1 (**B**), Occludin (**C**), ZO1 (**D**), Glut1 (**E**), LAT1 (**F**), Lrp1 (**G**), P-gp (**H**), BCRP **(I)**, Ager (**J**) and Cd31 (**K**) in the cortex of male and female mice at 3 and 10 months of age. (**L-R**) The mRNA levels of Cd31 **(L**), Cldn5 (**M**), Occludin (**N**), ZO1 (**O**), Glut1 (**P**), LAT1 (**Q**) and Lrp1 **(R**) in the brain microvascular samples of male and female mice at 3 and 10 months of age. BMEC: brain microvascular endothelial cell. Data are expressed as mean ± SEM. *n* = 5–6 per group; * *p* < 0.05, ** *p* < 0.01, **** *p* < 0.0001, compared with the 3-month male group; ^#^ *p* < 0.05, ^##^ *p* < 0.01, compared with the 10-month male group; ^$^ *p* < 0.05, ^$$^ *p* < 0.01, ^$$$$^*p* < 0.0001, compared with the 3-month female group

We respectively evaluated the expression differences of genes related to BBB permeability and transporter function between male and female mice in the two age groups (Fig. 1). The results showed that the cortex of in both age groups, the expression of tight junction protein Claudin5 (Cldn5) was significantly higher in male mice than in females (3-month-old: *p* = 0.0096; 10-month-old: *p* = 0.0191); in both male and female mice, the expression of Cldn5 also indicated an age-related downward trend (male: *p* = 0.0059; female: *p* < 0.0001) (Fig. 1A); whereas, no significant difference in the expression of tight junction protein Claudin1 (Cldn1) was evident regardless of the sex and age of the mice (Fig. 1B). Moreover, a significant sex difference was evident in the expression of the tight junction protein occludin, with the expression level in male mice significantly higher than that in females (3-month-old: *p* = 0.0016; 10-month-old: *p* = 0.0018), while only female mice reported a significant age-related decrease in the expression level of occludin (*p* = 0.0260) (Fig. 1C). Still, only the 3-month-old male mice reported a markedly higher expression of the membrane-associated guanylate kinases tight junction protein Tjp1 (also known as ZO1) when compared with the age-matched female counterparts (*p* = 0.0363) (Fig. 1D).

The analysis of BBB transporter-related genes revealed that in both age groups, compared with female counterparts, male mice reported a significantly higher expression of glucose transporter 1 (Glut1) and L-type amino acid transporter 1 (LAT1) in the cortex (3-month-old: *p* = 0.0116, *p* = 0.0246, respectively; 10-month-old mice: *p* = 0.0013, *p* = 0.0006, respectively) (Fig. 1E, F), indicating a significant sex difference. Meanwhile, compared with 3-month-old female mice, the age-matched male mice reported a noticeably higher expression of low-density lipoprotein receptor-related protein 1 (Lrp1) and permeability-glycoprotein (P-gp, encoded by the Abcb1 gene) (*p* = 0.0056, *p* = 0.0111, respectively), while no statistical difference was found between the 10-month-old male and female mice (Fig. 1G, H). Of note, compared with the 3-month-old female counterparts, their 10-month-old female mice reported a markedly lower expression of Glut1 but a higher expression of Lrp1 (*p* = 0.0097, *p* = 0.0095, respectively), while no difference was observed in the male mice (Fig. 1E, G). In addition, regardless of the sex, the 10-month-old mice displayed a significantly lower expression of P-gp and breast cancer resistance protein (Bcrp) than the 3-month-old counterparts (*p* < 0.0001for both) (Fig. 1H, I). Still, there were no significant sex differences and age-related changes in the expression of advanced glycosylation end product-specific receptor (Ager) (Fig. 1J). The expression of Cd31, a marker for vascular endothelial cells, was also examined in each group (Fig. 1K). We found that in both age groups, the mRNA level of Cd31 was significantly higher in male mice than in female mice (3-month-old: *p* = 0.0002; 10-month-old: *p* = 0.0079). Furthermore, Cd31 expression was slightly higher in the 10-month-old female mice than in the 3-month-old counterparts (*p* = 0.0440), whereas no significant changes were observed in the male mice.

To more intuitively observe the expression of these differentially expressed genes on the BBB, we extracted brain microvascular endothelial cells, which contained very low levels of neurons, astrocytes, and choroid plexus (Figure S3), and there was no significant difference in the expression of CD31 across the four groups (Fig. 1L). Several representative genes were detected by qPCR. Similar to cortical samples, in both age groups, male mice showed a higher expression of Cldn5 and occludin in brain microvascular extracts than female mice (3-month-old: *p* < 0.0001, *p* < 0.0001, respectively; 10-month-old mice: *p* < 0.0001, *p* = 0.0036, respectively) (Fig. 1M, N). Moreover, both male and female mice reported an age-related decrease in the expression of Cldn5 and ZO1 [male (3-month-old vs. 10-month-old): *p* = 0.0017, *p* = 0.0029, respectively; female (3-month-old vs. 10-month-old): *p* = 0.0034, *p* = 0.0213, respectively] (Fig. 1M, O), while only male mice showed an an age-related decrease in occludin (*p* < 0.0001) (Fig. 1N).

Regarding transporters, both male and female mice showed an age-related downregulation in Glut1 (male: *p* < 0.0001; female: *p* = 0.0021, respectively) (Fig. 1P). Furthermore, no sex-related differences in LAT1 expression were observed in either 3-month-old or 10-month-old mice, while an age-related decrease in its expression was observed only in female mice (*p* = 0.0070) (Fig. 1Q). Lrp expression was significantly higher in 3-month-old female mice than in male mice (*p* = 0.0027), while its expression was similar between 10-month-old female and male mice. Lrp1 expression was significantly lower in 10-month-old female mice than in 3-month-old female mice (*p* < 0.0001), while no age-related decrease was observed in male mice (Fig. 1R). These results highlight the sex differences and age-related characteristics of BBB permeability and the potential regulatory involvement of its transporter genes.

### Pericytes and basement membrane

**Fig. 2:**
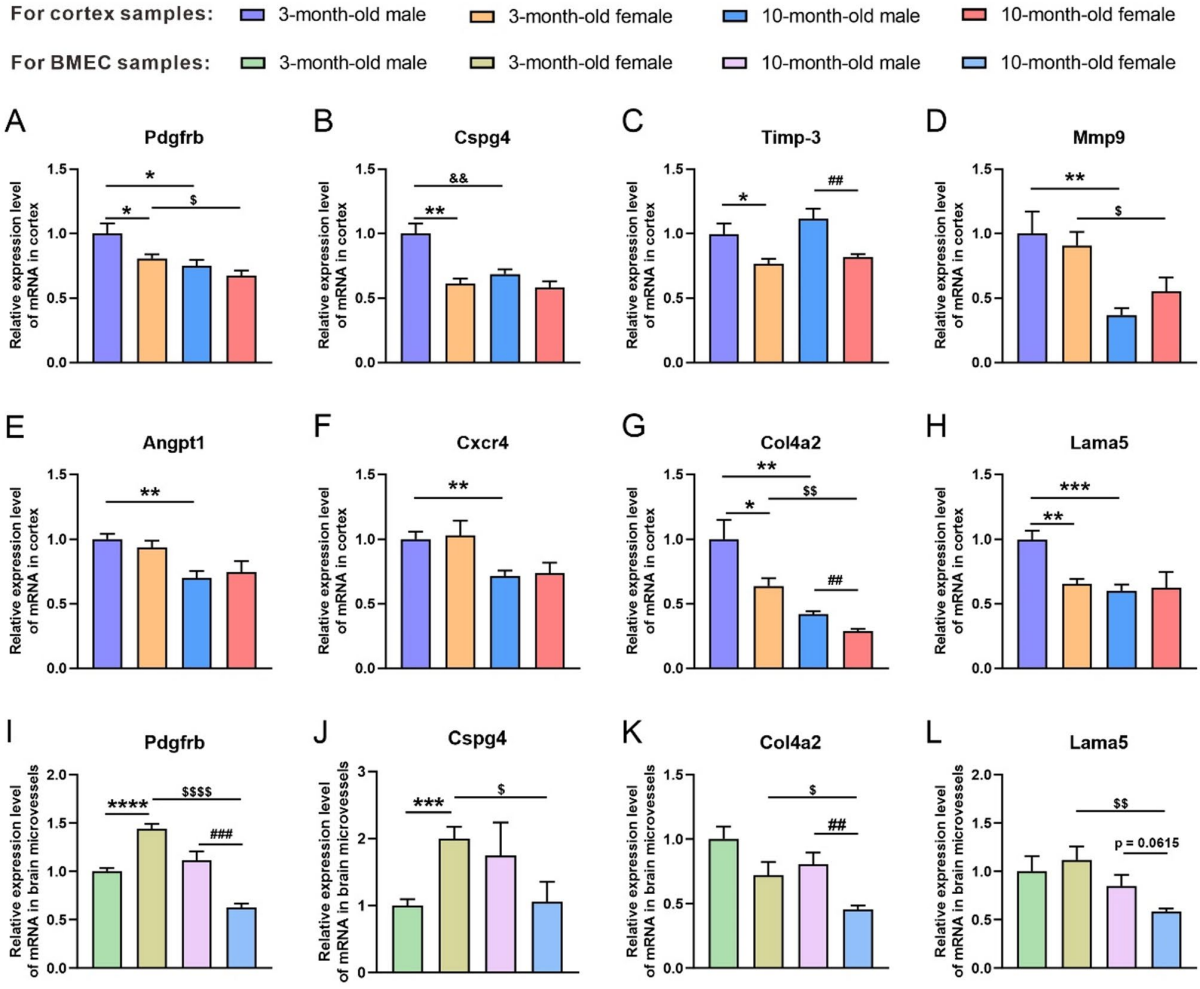
The mRNA levels of pericyte- and basement membrane-related genes in the cortex and brain microvascular samples of male and female mice at 3 and 10 months of age. (**A-H**) The mRNA levels of Pdgfrb (**A**), Cspg4 (**B**), Timp-3 (**C**), Mmp9 (**D**), Angpt1 (**E**), Cxcr4 (**F**), Col4a2 (**G**), and Lama5 (**H)** in the cortex of 3- and 10-month-old male and female mice. (**I-L**) The mRNA levels of Pdgfrb (**I**), Cspg4 (**J)**, Col4a2 (**K**) and Lama5 in the brain microvascular samples of male and female mice at 3 and 10 months of age. BMEC: brain microvascular endothelial cell. Data are expressed as mean ± SEM. *n* = 5–6 per group; * *p* < 0.05, ** *p* < 0.01, *** *p* < 0.001, **** *p* < 0.0001, compared with the 3-month male group; ^##^ *p* < 0.01, compared with the 10-month male group; ^$^ *p* < 0.05, ^$$^ *p* < 0.01, ^$$$$^*p* < 0.0001, compared with the 3-month female group

We further analyzed pericyte-related genes (Fig. 2). The results showed that in the cortex, compared with the 3-month-old female counterparts, the age-matched male mice reported a significant higher expression of platelet-derived growth factor receptor-b (Pdgfrb, the pericyte marker) and chondroitin sulfate proteoglycan 4 (Cspg4) (*p* = 0.0479, *p* = 0.0011, respectively), while no difference was evident between the 10-month-old male and female mice (Fig. 2A, B). In both age groups, there was a significant difference in the expression of tissue inhibitor of metalloproteinase-3 (Timp-3) between male and female mice (3-month-old: *p* = 0.0269; 10-month-old: *p* = 0.0041) (Fig. 2C). The longitudinal analysis showed that both male and female mice displayed a marked age-related decline in the expression of Pdgfrb and matrix metallopeptidase 9 (Mmp9) [male (3-month-old vs. 10-month-old): *p* = 0.0221, *p* = 0.0056, respectively; female (3-month-old vs. 10-month-old): *p* = 0.0278, *p* = 0.0463, respectively] (Fig. 2A, D). With the advancing age, the male mice showed a significant decrease in the expression of Cspg4, angiopoietin 1 (Angpt1), and the C-X-C motif chemokine receptor 4 (Cxcr4) (*p* = 0.0044, *p* = 0.0013, *p* = 0.0026, respectively) (Fig. 2B, E, F), suggesting that both age and sex may impact the pericyte development and functional regulation via the modulation of the expression of these genes.

Subsequently, we detected the important components of the basement membrane, collagen type IV α2 (Col4a2) and laminin alpha 5 (Lama5). The results revealed, in both age groups, a higher expression of Col4a2 in male mice than in female mice (3-month-old: *p* = 0.0492; 10-month-old: *p* = 0.0013), and an age-related decline for both sexes (male: *p* = 0.0034; female: *p* = 0.0019) (Fig. 2G). A sex difference was found in the expression of Lama5 in the 3-month-old mice (male vs. female: *p* = 0.0013), which disappeared in the 10-month-old mice. Compared with the 3-month-old mice, only the 10-month-old male mice reported a significant decrease in the expression of Lama5 (*p* = 0.0008), with the expression of Lama5 in female mice remaining at a low level (Fig. 2H). These results indicate that male mice have a greater basement membrane integrity than female mice and that the basement membrane reserve of male mice declines more significantly than that of female mice with age.

Similarly, we selected several representative genes and further examined them in brain microvascular endothelial cell samples. The results revealed that the expression of Pdgfrb was significantly higher in the 3-month-old female mice than in the age-matched male mice (*p* < 0.0001) and significantly lower in the 10-month-old female mice than in the age-matched male mice (*p* = 0.0006); furthermore, an age-related decrease in Pdgfrb expression was observed only in the female mice (*p* < 0.0001) (Fig. 2I). For the pericyte regulatory gene (Cspg4), the 3-month-old female mice reported a significantly higher expression than the age-matched male counterparts (*p* = 0.0009), while no sex difference was evident in the 10-month-old mice; and an age-related downregulation was observed only in the female mice (*p* = 0.0260) (Fig. 2J).

In addition, components of the basement membrane, Col4a2 and Lama5, were more significantly expressed in the 10-month-old males than in the age-matched females (*p* = 0.0053; *p* = 0.0615, respectively), while only female mice reported an age-related downregulation in both components (*p* = 0.0405; *p* = 0.0059, respectively) (Fig. 2K, L). Taken together, despite the inconsistency between the results of the brain microvascular samples and those of the total cortex, the findings still evidence obvious sex differences and age-related decrease in pericytes and basement membrane reserves.

### Endothelial glycocalyx

**Fig. 3:**
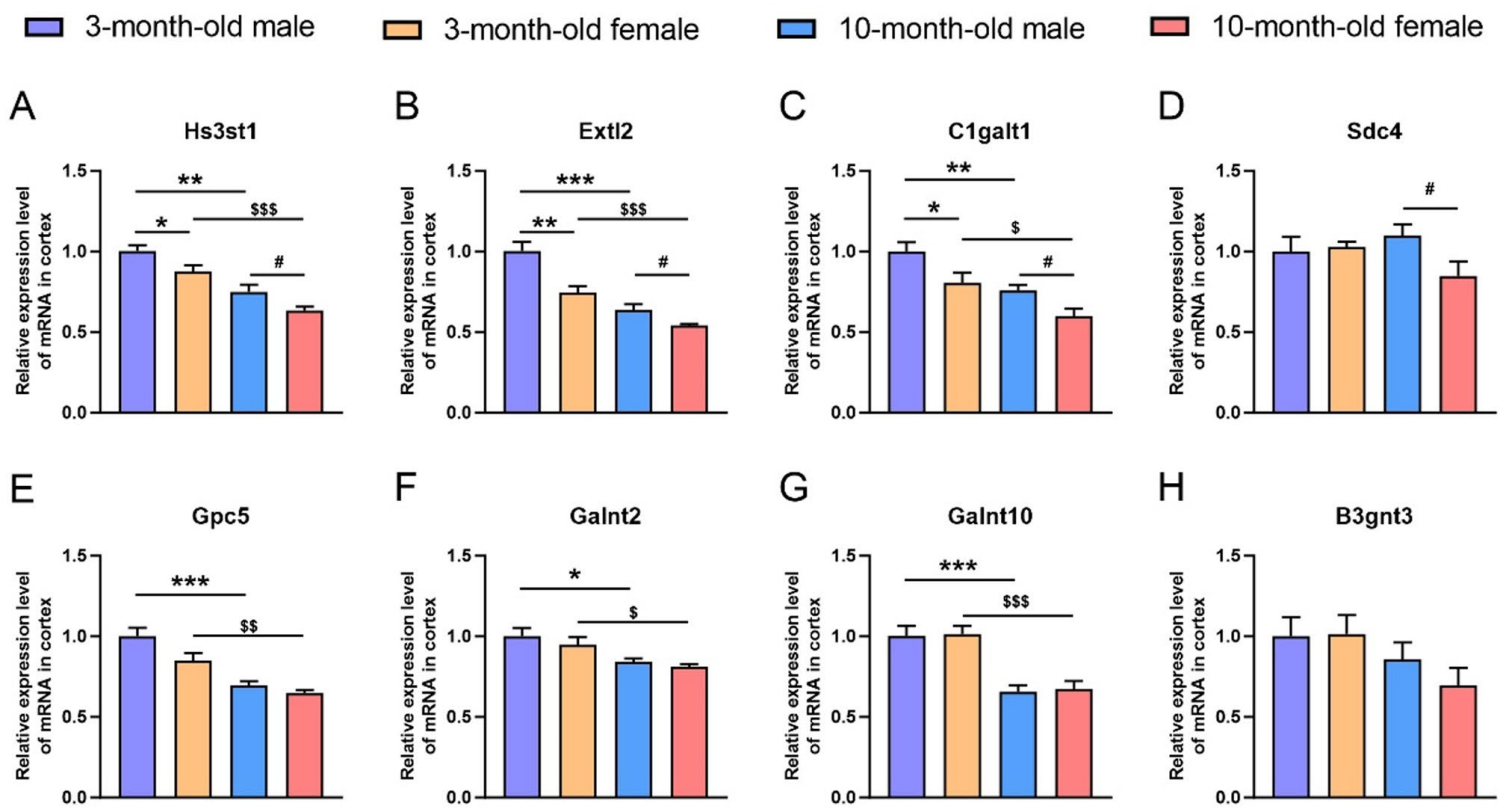
The mRNA levels of glycocalyx-related genes in the cortex of male and female mice at 3 and 10 months of age. (**A-H**) The mRNA levels of Hs3st1 (**A**), Extl2 (**B**), C1galt1 (**C**), Sdc4 (**D**), Gpc5 (**E**), Galnt2 (**F**), Galnt10 (**G**), and B3gnt3 (**H**) in the cortex of male and female mice at 3 and 10 months of age. Data are expressed as mean ± SEM. *n* = 6 per group; * *p* < 0.05, ** *p* < 0.01, *** *p* < 0.001, compared with the 3-month male group; ^#^ *p* < 0.05, compared with the 10-month male group; ^$^ *p* < 0.05, ^$$^ *p* < 0.01, ^$$$^ *p* < 0.001, compared with the 3-month female group

We then explored the expression characteristics of endothelial glycocalyx-related genes in the cortex of these groups (Fig. 3). The results showed that in both age groups, compared with the female counterparts, the male mice reported a significantly higher expression of heparan sulfate 3-O-sulfotransferase 1 (Hs3st1), exostosin-like glycosyltransferase 2 (Extl2), and core 1 synthase, glycoprotein-N-acetylgalactosamine 3-beta-galactosyltransferase 1 (C1galt1) (3-month-old: *p* = 0.0381, *p* = 0.0040, *p* = 0.0469, respectively; 10-month-old: *p* = 0.0471, *p* = 0.0227, *p* = 0.0204, respectively) (Fig. 3A-C), while only the 10-month-old male mice displayed a higher expression of syndecan 4 (Sdc4) than the age-matched females (*p* = 0.0480) (Fig. 3D). In addition, in both male and female mice, an age-related change was found in Hs3st1 (male: *p* = 0.0015; female: *p* = 0.0003), Extl2 (male: *p* = 0.0003; female: *p* = 0.0005), C1galt1 (male: *p* = 0.0053; female: *p* = 0.0245), glypican 5 (Gpc5) (male: *p* = 0.0005; female: *p* = 0.0029), O-glycosylating enzyme Galnt2 (male: *p* = 0.0175; female: *p* = 0.0225), and Galnt10 (*p* = 0.0009 for both), suggesting that the regulation of the expression of glycocalyx-related genes may be closely related to sex and age (Fig. 3A-C, E-G). Our results indicate that glycocalyx integrity deteriorates with age in mice, which fares worse in female mice than in males.

### Vascular hypoxia, inflammation and others

**Fig. 4:**
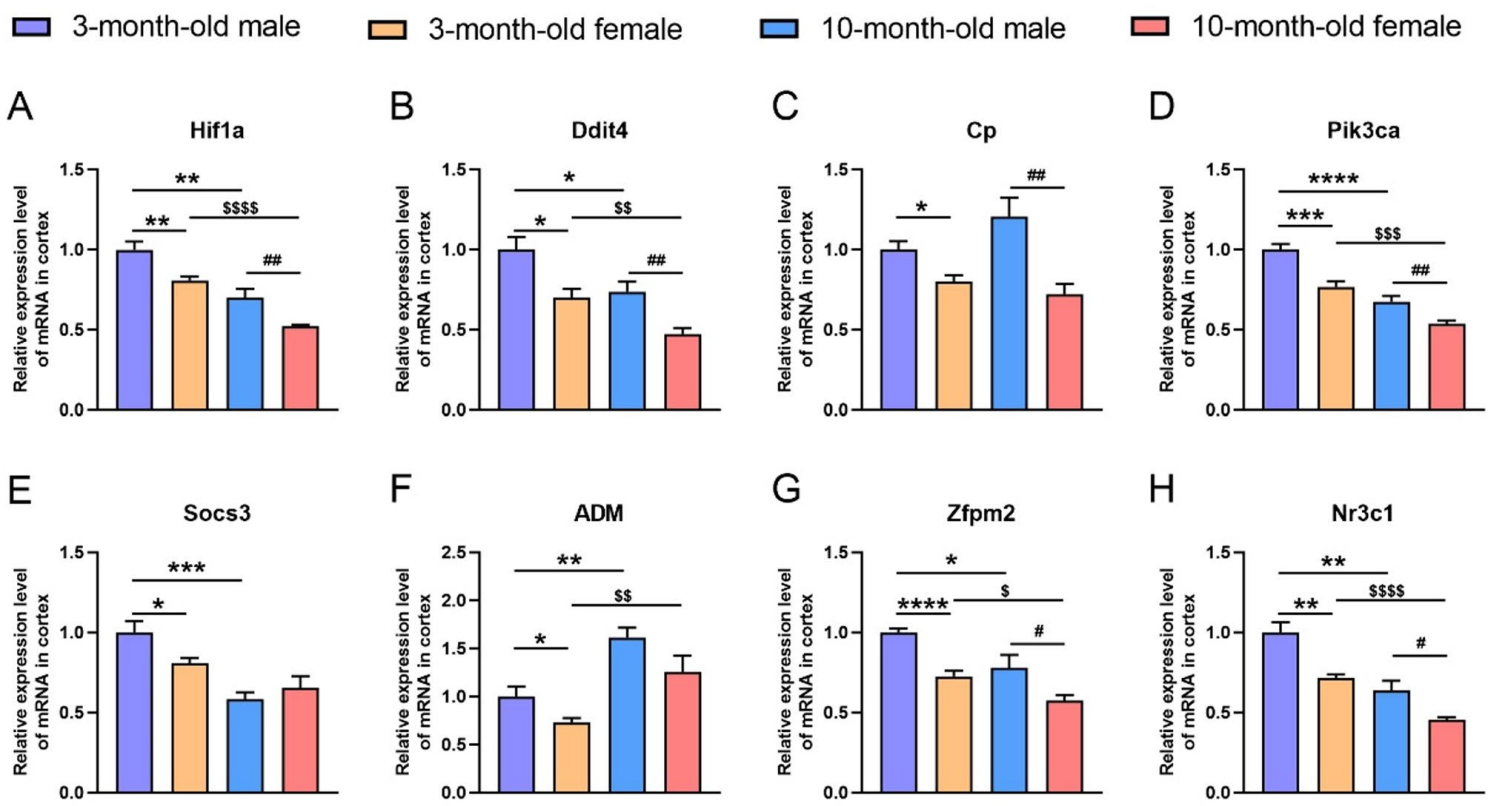
The mRNA levels of vascular hypoxia, inflammation-related genes and other regulatory genes in the cortex of male and female mice at 3 and 10 months of age. (**A-H**) The mRNA levels of Hif1a (**A**), Ddit4 (**B**), Cp (**C**), Pik3ca (**D**), Socs3 (**E**), ADM (**F**), Zfpm2 (**G**), and Nr3c1 (**H**) in the cortex of male and female mice at 3 and 10 months of age. Data are expressed as mean ± SEM. *n* = 6 per group; * *p* < 0.05, ** *p* < 0.01, *** *p* < 0.001, **** *p* < 0.0001, compared with the 3-month male group; ^#^ *p* < 0.05, ^##^ *p* < 0.01, compared with the 10-month male group; ^$^ *p* < 0.05, ^$$^ *p* < 0.01, ^$$$^ *p* < 0.001, ^$$$$^*p* < 0.0001, compared with the 3-month female group

Vascular hypoxia and inflammation can also affect the function of the BBB. We further analyzed the expression of the related genes (Fig. 4). The results showed that in the cortex of both age groups, compared with the female counterparts, the male mice reported a significantly higher expression of hypoxia inducible factor 1α (Hif1α) (3-month-old: *p* = 0.0085; 10-month-old: *p* = 0.0077), DNA-damage-inducible transcript 4 (Ddit4) (3-month-old: *p* = 0.0118; 10-month-old: *p* = 0.0057), and ceruloplasmin (Cp) (3-month-old: *p* = 0.0140; 10-month-old: *p* = 0.0047) (Fig. 4A-C); and both male and female mice displayed an age-related decrease in the expression of Hif1a (male: *p* = 0.0028; female: *p* < 0.0001) and Ddit4 (male: *p* = 0.0289; female: *p* = 0.0063) (Fig. 4A, B).

Next, we tested two genes that are related to the inflammation in the vascular microenvironment. The results revealed a higher expression of PI3K catalytic subunit alpha (Pik3ca) in the male mice than in the female counterparts in both age groups (3-month-old: *p* = 0.0009; 10-month-old: *p* = 0.0085) and an age-related decline in its expression in both male and female mice (male: *p* < 0.0001; female: *p* = 0.0003) (Fig. 4D). Moreover, a sex difference in the expression of suppressor of cytokine signaling 3 (Socs3) was observed in the 3-month-old male mice when compared with the age-matched females (*p* = 0.0374), which disappeared in the 10-month-old group; an age-related decrease in the expression of Socs3 was found in the 10-month-old male mice (*p* = 0.0006) (Fig. 4E). Taken together, these results reveal that there are sex- and age-related differences in the specific patterns of gene expression regulation during vascular hypoxia and inflammatory response.

We also examined genes that have indirect effects on BBB permeability, such as adrenomedullin (Adm), Zinc finger protein FOG family member 2 (Zfpm2), and nuclear receptor subfamily 3 group C member 1 (Nr3c1). The former strengthens tight junctions by regulating elevated intercellular cAMP concentrations; the latter two regulate brain vascular development and transporter expression, respectively. The results reported a higher expression of Adm in the 3-month-old male mice than in the age-matched females (*p* = 0.0423), no sex difference in the 10-month-old mice, and an age-related difference in both male and female mice, with a higher expression of Adm in the 10-month-old mice than in the 3-month-old counterparts (male: *p* = 0.0020; female: *p* = 0.0095) (Fig. 4F). Furthermore, sex- and age-related differences in the expression of Zfpm2 and Nr3c1 were observed, with a higher expression evident in the male mice than in the female counterparts (Zfpm2: *p* < 0.0001, *p* = 0.0371; Nr3c1: *p* = 0.0021, *p* = 0.0158, respectively) and in the 3-month-olds than in the 10-month-olds (Zfpm2: *p* = 0.0248, *p* = 0.0116; Nr3c1: *p* = 0.0023, *p* < 0.0001, respectively) (Fig. 4G, H).

## Discussion

In this study, we systematically analyzed the expression profiles of genes related to BBB function, with a particular focus on the impact of sex and age differences. Our results revealed that in a physiological context, there are sex differences and age-related changes in the expression of genes related to BBB molecular structure, transporters, and vascular pathology. These findings not only provide a new perspective for understanding BBB heterogeneity, but also lay a molecular foundation for sex- and age-specific treatment strategies for neurological diseases.

Brain microvessels are the anatomical foundation of the BBB. In the cortex, we found that the expression of the brain microvascular marker, Cd31, was significantly higher in males than in females, a trend also observed in several other genes we examined. We speculate that the sex-specific expression differences in these genes may be attributed to the higher cortical vascular density in male mice when compared with the females. In isolated brain microvascular samples, the expression patterns of most genes examined were similar to those in the cortex, indicating significant differences not only in the number of brain microvessels but also in the expression of these genes per microvessel. These two factors may collectively contribute to the higher expression of BBB-related genes in the cortex of the males.

The structure of the BBB ​​has an important influence on its permeability and is the physical basis for its function. As a physical barrier between brain microvascular endothelial cells, tight junctions prevent solutes and water from freely passing through the paracellular space between epithelial or endothelial cell sheets and play a key role in maintaining cell polarity and signal transduction. The integrity of the barrier is maintained by, two core proteins of tight junctions, Cldn5 and occludin, and the auxiliary protein, ZO1 [39]. Our study found that the expression of these three genes in the cortex and brain microvessels was different between male and female mice, with a significantly higher expression in male mice than in female mice, indicating that under normal physiological conditions, the structure of tight junctions in male mice is more intact and stable. Such a difference is typically manifested in certain disease scenarios. For example, depression is more prevalent in women than in men [40–42]. More telling evidence reveals that chronic stressors can lead to the loss of Cldn5 in the prefrontal cortex (PFC) of female mice, thereby changing the integrity of the BBB. Similar changes have been found in the autopsy of brain tissues from female patients with depression [43], which has not been observed in the PFC of male mice [44], suggesting that the effects of chronic stress and depression on the neurovascular system are sex-specific. In another example, 24 h after traumatic brain injury, the accumulation of macromolecular tracer horseradish peroxidase and nanoparticles was significantly higher in female mice than in male mice, indicating sex-dependent differences in BBB permeability [45]. Similar sex differences in BBB permeability have also been reported found in the bicuculline-induced Wistar rat epilepsy model, with the extravasation of BBB tracer Evans blue significantly pronounced in the brain tissue of female rats than in male rats [46].

In addition, the current study found an age-related decrease in both Cldn5, occludin and ZO1, suggesting that with the advancing age, the barrier function of the BBB decreases and the permeability increases, which is particularly evident in the development of AD. In the available literature, BBB disruption has been recognized as an early indicator of cognitive dysfunction in humans, for some individuals with early cognitive dysfunction may present cerebral capillary damage and hippocampal BBB disruption without the presence of amyloid-beta peptides and/or tau biomarker changes that are typical in AD [47]. Consistently, previous studies have documented a marked increase in BBB permeability in the hippocampus of patients with moderate cognitive impairment (MCI) when compared with healthy controls by contrast-enhanced MRI [48, 49], and age-dependent BBB disruption in the hippocampus of patients with AD by postmortem tissue analysis [50].

Transporters are key functional components of the BBB, regulating the balance of substances in the brain through active transport or facilitative diffusion. As the most abundant transporter on the BBB, the glucose transporter Glut1 is highly specific in BBB endothelial cells and is responsible for unidirectional transport of glucose from blood to brain tissue to maintain brain energy supply [51]. LAT1 can transport essential amino acids (such as leucine and phenylalanine) and some drugs (such as levodopa), and is crucial for the transport of neurotransmitter precursors in the brain [52]. In our study, we found a sex difference in the expression of Glut1 and LAT1 in both age groups, with a significantly higher expression in male mice than in female mice, suggesting a higher demand of energy and protein supply in the males.

As an important component of the BBB, the efflux transporter P-gp limits the accumulation of many compounds in the brain. It can facilitate the excretion of many drugs from the brain tissues [53]. Studies show that P-gp can interact with some opioids in vitro and in vivo and that P-gp-mediated opioid efflux across the BBB may affect the onset, intensity, and duration of analgesic response [54, 55]. Our study found that the expression of P-gp in male mice at 3 months of age was significantly higher than that of age-matched female mice, which is consistent with the previous findings [56]. Another important efflux transporter of the BBB, BCRP can pump a variety of drugs, endogenous metabolites, neurotoxins, etc., from brain microvascular endothelial cells into the blood, thereby limiting the entry of these substances into the brain to reach the lesion sites [57]. Typical examples include chemotherapy drugs, antihistamines, statins, etc. The presence of BCRP may account for the poor efficacy of some drug treatments for brain diseases. Still, as a dual transporter, LRP1 in brain endothelial cells can bind to Aβ and mediate the clearance of Aβ from the brain to the blood. The damage of LRP1 may aggravate the progression of AD [58, 59]. Our experimental results in the brain microvessels showed that with the advancing age, the level of LRP1 declined more significantly in female mice than in male mice, which may shed some lights on the high prevalence of AD in female patients.

In addition, our study found a significant age-related downregulation of the expression of efflux transporters, BCRP and P-gp in both sexes, which signifies that the barrier effect of the BBB against certain neurotoxins, metabolic wastes, and drugs declines with age. The reduced expression of BCRP and P-gp may impair the clearance of harmful substances, making nerve cells more vulnerable to damage, thereby increasing the risk of diseases such as AD and PD. In turn, the clearance impairment can increase the accumulation of drugs in the brain and the risk of neurotoxicity, so, with these drugs, the elderly is more likely to experience adverse reactions in the CNS.

Our results also showed that the expression of Glut1 in the brain microvessels decreased in both male and female mice with the advancing age, reflecting the reduced transport of glucose into the brain, which is also one of the early pathological characteristics of AD [60], as the reduction of Glut1 can exacerbate the vascular and neurological dysfunction and degeneration in AD pathology [14].

Pericytes are in the basement membrane of brain microvessels, wrapping around endothelial cells through long synaptic extensions to form a “net-like” structure. They directly contact endothelial cells through N-cadherin and connexins [61, 62]. Recent studies have shown that pericytes are crucial not only for the formation of the BBB during embryonic development [19, 63], but also for the maintenance of the BBB in adulthood and post-injury repair [64, 65]. Pericyte deficiency has been reported to increase the permeability of the BBB to water and a range of low-molecular-mass and high-molecular-mass tracers [64] and pericyte damage is associated with many CNS diseases, including AD [66–69], aging [50], stroke [70], PD [71], amyotrophic lateral sclerosis [67], and diabetic retinopathy [72].

As a surface receptor of pericytes, PDGFRβis considered as a marker of pericytes. Pericytes regulate microvascular maturation through the PDGF-β/PDGFR-β signaling pathway, and stabilize endothelial cell tight junctions by secreting Angpt1 [20], counteracting the leakage caused by inflammatory factors [73]. The defects of the PDGF-B/PDGFRβ pathway can cause pericyte loss and the formation of microvascular tumors in mice [74]. In turn, pericyte loss or dedifferentiation can cause abnormal BBB permeability and promote neuroinflammatory cascades. As a key regulatory factor in pericyte development and migration, Cspg4, also known as neuron-glial antigen 2 (NG2), is mainly expressed in oligodendrocyte precursor cells and pericytes [75]. It participates in the formation of new blood vessels and vascular remodeling during the BBB formation stage [76]. NG2 deficiency can lead to insufficient pericyte coverage and increase the permeability of the BBB to macromolecules (such as albumin) [77]. Our results also reported an age-related decrease in the expression of Pdgfrb, NG2, and Angpt1 in the male mice, indicating that the function of pericytes in the male mice decreases with age, while that of female mice remains relatively stable despite the advancing age. In the brain microvascular samples, however, the expression of Pdgfrb and NG2 was significantly higher in the 3-month-old female mice than in the age-matched males. We speculate that this observation may be attributed to the fact that the high pericyte density in young female mice may enhance BBB stability and maintain barrier function, supporting the relatively more active barrier function of the youth.

Glycocalyx is a complex polysaccharide-protein network structure covering the surface of endothelial cells. It is mainly composed of proteoglycans, glycoproteins and glycolipids. It maintains the selective permeability of the BBB through charge barriers and mechanical sensing. The removal of any major component will destroy the glycocalyx layer and increase vascular permeability [23, 78]. Heparan sulfate proteoglycans are the main proteoglycans that make up the glycocalyx, mainly including syndecans (SDCs) and glypicans (GPCs), and the metabolism of heparan sulfate proteoglycans mainly involves genes such as Sdc4, Hs3st1, Extl2 and Gpc5. As heparan sulfate proteoglycans play a role in regulating vascular permeability, microcirculatory tension, leukocyte adhesion and platelet hemostasis [79], their degradation can directly lead to barrier leakage of the BBB and infiltration of inflammatory factors. The degradation products can peel off from the cell surface and enter the circulatory system. Therefore, the changes in the concentration of these degradation products can be used as indicators to assess the degree of glycocalyx damage and disease status [80]. Significant changes in HSPG levels have been reported in cardiovascular diseases [81–83], sepsis [79], diabetes [84, 85], and neurodegenerative diseases [26]. Our results showed that the expression of Hs3st1 and Extl2 in male mice at 3 and 10 months of age was significantly higher than that in female mice, and the expression of Gpc5 in male mice at 10 months of age was higher than that in female mice, indicating that male mice metabolize more heparan sulfate proteoglycans than female mice and have a poorer glycocalyx integrity. In addition, compared with the 3-month-old mice, the 10-month-old mice reported a significantly lower expression of the above genes, indicating that the glycocalyx integrity of the 10-month-old mice is better retained than that of the 3-month-old mice, which is not consistent with the available reports. A previous experiment compared the glycocalyx of the 3-month-old and 19-month-old mice and found that the levels of Sdc4, Hs3st1, Extl2 and Gpc5 genes in the 19-month-old mice were significantly higher than those in the 3-month-old mice [26]. The inconsistency may be attributed to the difference in age (10 months old vs. 19 months old) and the relatively stable glycocalyx function at 10 months of age. With the further increase in age, the integrity of the glycocalyx is significantly downregulated.

N- and O-linked glycans are the two most prominent forms of glycosylation. Among them, O-GalNAc glycans are the most important sugars in the glycocalyx [86]. The Galnt family proteins are enzymes that regulate the initial step in mucin O-glycan synthesis. In the process, these enzymes covalently bind the GalNAc group of the glycoside donor UDP-GalNAc to the side chain hydroxyl of the serine or threonine residue of the protein to form a GalNAc-α-Ser/Thr structure, also known as the Tn antigen structure [87, 88]; C1galt1 catalyzes the addition of galactose to the Tn antigen (GalNAc) to form the core 1 structure (T antigen); and B3gnt3 adds GalNAc to the core 1 structure to mediate sugar chain extension. These genes work together to complete the biosynthesis of O-GalNAc glycans [89]. O-GalNAc glycans are important components of the glycocalyx, and, together with glycoproteins, they build the physical barrier of the glycocalyx, mediate intercellular communication, and regulate permeability. In our study, compared with the 3-month-old mice, both the 10-month-old male and female mice showed a significant downregulation of Galnt10, Galnt2 and C1galt1, indicating a decline in the initiation and elongation of O-GalNAc glycans, which also means that the structure of the glycocalyx is affected. This finding is consistent with available reports that the downregulation of the expression of the above genes in the brain endothelial cells of old mice is associated with a significant reduction in the thickness and area of ​​the glycocalyx layer, signifying that the destruction of the glycocalyx structure can lead to increased BBB permeability and even cerebral hemorrhage [26]. Moreover, existent studies show that the knockout of C1galt1 can cause fatal embryonic hemorrhage due to defective angiogenesis [90] and that an AAV-mediated overexpression of C1galt1 and B3gnt3 can reduce BBB permeability and thus improve brain health by reducing the expression of neuroinflammatory markers and ameliorating cognitive function [26]. Late-stage AD patients also show reduced T-synthetase activity and defective galactosylation [91]. In the current study, compared with the male mice of both age groups, the expression of C1galt1 in female mice was significantly lower, indicating that the galactosylation of female mice is worse than that of male mice. Our results demonstrate sex-specific and age-related changes in glycocalyx function and confirm the importance of glycocalyx for BBB integrity.

Lama5 and Col4a2 are key components of the basement membrane, providing structural support and stability for the BBB. Under homeostatic conditions, the loss of endothelial Lama5 does not affect cerebrovascular structure and BBB integrity, whereas after intracranial hemorrhage, the conditional knockout of endothelial-specific Lama5 may increase BBB permeability, vascular rupture, and inflammatory cell infiltration, and impair neurological function [92]. In our results, the male mice had relatively higher Lama5 and Col4a2 expression than the female mice, thus providing a better basement membrane structure for the BBB and curtailing the invasion of harmful substances from the outside world. We also found that the expression of Lama5 and Col4a2 in the 10-month-old mice was significantly reduced, indicating a gradual impairment in the basement membrane structure, an increase in the BBB permeability, and the probable entry of toxic substances and immune cells in the blood into the brain tissue, which ultimately results in aggravated inflammation and nerve cell damage and accelerates the progression of neurological diseases and aging.

Vascular aging and hypoxia can induce the accumulation of reactive oxygen species (ROS) in the endothelial cells and mitochondrial dysfunction, weaken the expression of tight junction proteins ZO-1 and occludin, and increase the leakage of BBB in aged mice [93, 94]. Meanwhile, they downregulate the activity of efflux transporters such as P-gp, exacerbating the retention of neurotoxic substances [31]. Hypoxia-induced neuroinflammation activates microglia and astrocytes, releasing inflammatory factors and free radicals, which continue to damage the matrix and neurovascular tissues over time. With age, the integrity of the BBB decreases and is more susceptible to stress factors such as hypoxia [95], thus forming a vicious cycle. Under hypoxic conditions, Hif1a is activated and can regulate the expression of multiple genes to adapt to the hypoxic environment. However, overactivated Hif1a may disrupt the normal function of endothelial cells, affect the expression of tight junction proteins, and increase the permeability of the BBB [29, 30, 96]. Ddit4 is an important gene for the conduction and regulation of Hif1a and mechanistic target of rapamycin kinase (mTOR) signaling pathways. It plays a role in the response of cells to adverse environments such as hypoxia and energy stress, and can regulate the metabolic process and autophagy of cells [97, 98], indirectly affecting the tight junctions and permeability of cells. In our results, the expression of Hif1a and Ddit4 was significantly higher in male mice than in female mice and in the young mice than in the old counterparts, indicating that under physiological conditions, male mice have better hypoxia tolerance and metabolic capacity than female mice and that young individuals have stronger regulatory ability in the face of hypoxia and the reduction of Hif1a in old mice may aggravate the brain energy metabolism.

CP is a multi-copper iron oxidase that is mainly synthesized by astrocytes and brain microvascular endothelial cells in the CNS. CP can catalyze the oxidation of Fe²⁺ to Fe³⁺, promote the binding of iron ions to transferrin, and reduce the accumulation of free Fe²⁺ in cells, thus avoiding the production of ROS by the Fenton reaction and protecting cells from oxidative damage [99]. Brain iron accumulation and reduced CP activity have been shown to be associated with neurodegenerative diseases. Our data showed that the Cp expression in female mice at 3 and 10 months of age was significantly lower than that in males, indicating that female mice run a higher risk of oxidative stress and male mice have a more efficient iron removal, thereby reducing the risk of oxidative damage.

The inflammatory processes can promote BBB disruption, including pyroptotic endothelia, abnormal appearance of tight junctions, and vasculature detachment from the basement membrane [37]. It has been reported that a variety of chemical drugs can enhance BBB integrity during ischemia/reperfusion injury by regulating the PI3K/Akt/mTOR signaling pathway [100–102]. As the core catalytic subunit of PI3K, Pik3ca indirectly participates in regulating BBB integrity by activating the AKT/mTOR pathway [103]. Socs3 is a negative regulator of the Janus kinase/signal transducer and activator of the transcription (JAK/STAT) pathway. It inhibits the signaling of proinflammatory cytokines (such as interleukin-6 and tumor necrosis factor -α) by interfering with the STAT pathway, promotes the transition of microglia from the M1 phenotype to the M2 phenotype, and exerts an anti-inflammatory effect, thereby protecting the integrity of the blood-spinal cord barrier [104, 105]. In the current study, there were sex differences and age-related decline in the expression of Pik3ca and Socs3, indicating that the brain of young individuals, especially for males, relies more on the PI3K-AKT pathway to maintain plasticity and metabolic activity. The high expression of Socs3 may protect young individuals from excessive inflammation or metabolic stress; the decline in expression after middle age may be related to neurological decline and the initiation of chronic inflammation.

In addition, there are some genes, such as Adm, which is a vasoactive peptide that promotes the proliferation and migration of endothelial cells, thereby inducing angiogenesis. ADM activates the adenylyl cyclase/cAMP/PKA signaling pathway, which can elevate the intercellular cAMP concentration to increase Cldn5 expression and tight junctions, thus reducing brain endothelial permeability and ultimately maintaining the barrier function of the BBB [106, 107]. ADM has also been found to exert a protective effect against traumatic brain injury [108], ischemic brain injury [109], and depression [110]. In our results, male mice reported a relatively higher expression of Adm than female mice, which indicates a stronger dilation ability and angiogenesis potential in cerebral blood vessels of the male mice and a more stable blood perfusion under physiological conditions. With the advancing age, the expression of Adm in male and female mice increases. However, excessive ADM expression may disrupt vascular homeostasis and increase BBB instability.

Zfpm2, a transcriptional regulator, participates in cell differentiation and development and plays an important role in cerebrovascular development and neural tissue formation [111, 112]. On the other hand, Nr3c1 is a nuclear receptor and a glucocorticoid receptor. Studies have reported that Nr3c1 agonist glucocorticoids can significantly increase the activity and protein expression of BBB transporters, ABCG2 and ABCC5 [113]. In the current study, there were obvious sex differences and age-related decreases in the expression of Zfpm2 and Nr3c1 genes. We hypothesize that the downregulation of these two genes may induce poor angiogenesis and a reduction in BBB transporters, resulting in an insufficient cerebrovascular system in female mice to maintain normal brain responses and accordingly an increase in pathological susceptibility.

Clinically, the incidence of neurodegenerative diseases (such as AD) and neuropsychiatric diseases (such as depression and anxiety) is also significantly higher in females than in males [114–116]. Based on our experimental results, there was obvious sexual dimorphism in the structure and function of the BBB, which may be closely related to the regulation of sex hormones. Available studies evidence that cerebral blood vessels are the non-reproductive target organs of sex steroids and that gonadal hormones as fat-soluble small molecules can diffuse into the brain through the BBB and activate the downstream signaling pathways after binding to corresponding receptors [117]. However, existent studies have reported that both estrogen and androgen have beneficial effects in maintaining the integrity of the BBB [33] and that androgen deprivation therapy can cause complex changes in the immune and inflammatory response in the peripheral blood and brain, destroying the integrity of the BBB and promoting the infiltration of immune cells into the brain [118]. Other studies have documented that a chronic testosterone depletion may increase the permeability of the BBB to endogenous immunoglobulins, which is associated with a decrease in Cldn5 and ZO1, accompanied by an increased activation of astrocytes and microglia and the release of inflammatory factors, while a testosterone supplementation can restore the selective permeability of the BBB and the integrity of tight junctions [34]. Still others reveal that physiological concentrations of dehydroepiandrosterone sulfate can stimulate the expression of ZO1 and Cldn3 and promote the formation of tight junctions between adjacent cells [119] and that androgens not only affect the tight junctions of the BBB but also regulate the caveolin pathway and impact organic anion transporter 3, thus regulating BBB permeability [120]. Therefore, androgens play an important role in maintaining the integrity of the BBB in male mice.

Meanwhile, cumulative studies also demonstrate the beneficial effect of estrogen on the integrity of the BBB in female rodents. For example, 17β-estradiol can restore the integrity of the BBB in bilaterally ovariectomized rats [35] and increase the expression of Cldn5 in endothelial cells [36]; and estrogen can protect the BBB from damage caused by short-term inflammation [121]. However, the overall results of our study showed that compared with female counterparts, male mice reported a higher expression of BBB-related genes. The possible explanation for this discrepancy may lie in that the “high baseline expression” observed in males is intrinsic in a physiological context and may have a stronger basic regulatory advantage. Most of the available reports focus on the effects of sex hormones on BBB integrity and function under pathological conditions and fail to compare the BBB integrity between male and female mice under physiological conditions. In addition, in our results, the expression of genes in 3-month-old male mice was higher than that of 10-month-old male mice, suggesting a stronger BBB homeostasis, while the expression of these genes in 10-month-old male mice decreased with the increase of age, indicating a degeneration of the BBB.

## Limitations

While this study systematically reveals the sex dimorphism and age-related changes in the expression of BBB-related genes under physiological conditions, several limitations remain as follows. First, the study only detected gene expression at the mRNA level, lacking protein-level verification and functional experimental support. Although changes in mRNA can reflect alterations in genetic transcriptional activity, providing important insights into the molecular mechanisms underlying BBB structure and function, the mRNA expression levels are not entirely equivalent to protein levels, as the process from transcription to translation is subject to multiple regulatory processes, potentially leading to discrepancies between the two. Furthermore, mRNA levels cannot reflect the protein function, which depends not only on its expression level but also on post-translational modifications [122, 123]. Even with consistent trends in mRNA and protein levels, the regulation of protein activity can still lead to a disconnection between function and expression, a dimension not addressed by this study. The mRNA differences observed in this study only suggest potential molecular regulatory trends but cannot directly prove whether these genes affect BBB function (e.g., decreased barrier integrity or abnormal transport function) through changes in protein levels. Therefore, future studies are needed to further examine the protein expression and post-translational modification levels of key genes and, in conjunction with BBB functional assays, correlate these molecular expression changes with the overall barrier function to pinpoint the mechanisms regulating BBB function in relation to age and sex.

For another, mouse models are classic tools for studying BBB structure and function. The core components of their functional BBB are fundamentally similar to those of humans, making it easy to manipulate variables through age or sex stratification, providing a practical experimental system for analyzing BBB changes due to sex difference and aging. However, significant species-specificity in the BBB exists between the rodents and primates [124], potentially limiting the direct extrapolation of the findings to humans, although the age- and sex-related differences in the mRNA expression of BBB-related genes observed in the mouse models may provide foundational data for understanding the molecular regulatory trends of mammalian BBB aging. Structurally, the human brain NVU is more complex than that of the mice; molecularly, differences also exist in the expression patterns or regulatory mechanisms of homologous genes in the BBB of humans and mice [125]. More importantly, the aging rates of mice and humans are not completely identical [126]. Therefore, future studies should compare age- and sex-related differences in homologous genes in the BBB of humans and mice using in vitro models with human brain microvascular endothelial cells or by analyzing sequencing data from human brain samples, thereby more accurately extrapolating animal findings to humans.

Finally, the C57BL/6J mice used in this study are one of the most commonly used inbred strains in neurobiological research. Their genetic background is stable and rich data on the BBB are available, facilitating the comparison of our results with those of existing studies. However, it is important to note that there are differences in protein expression levels of transporters, receptors and marker proteins in the BBB among different mouse strains [127], potentially limiting the direct generalizability of our findings to other strains. Nevertheless, as an internationally recognized standard strain, the C57BL/6J data remain valuable as a reference.

## Perspectives and significance

Sex- and age-related differences in BBB are not only a core to understanding the mechanisms of physiological homeostasis in the nervous system, but also a key link connecting basic scientific research and clinical application transformation. This research holds great scientific value and clinical guiding significance for explaining the sex/age difference in the incidence and prognosis of nervous system diseases, and implementation of individualized treatment. By revealing the biological essence behind individual differences, this study will provide new research perspectives and theoretical foundations for the development of precision medicine, ultimately facilitating a safer and more efficient prevention of and the development of treatment strategies for nervous system diseases.

## Conclusion

Under physiological conditions, there are sex dimorphism and age-related changes in the expression of BBB-related genes, including tight junctions, transporter systems, pericyte functional molecules, and vascular pathology. The differences may impact the integrity and functions of the BBB. These findings may provide theoretical insights into the pathogenesis of neurological diseases and facilitate the design of sex/age-specific treatment strategies.

## Supplementary Information

Supplementary Material 1.

Supplementary Material 2.

Supplementary Material 3.

Supplementary Material 4.

Supplementary Material 5.

## Data Availability

No datasets were generated or analysed during the current study.

## Abbreviations

AD: Alzheimer’s disease
Adm: Adrenomedullin
Ager: Advanced glycosylation end product-specific receptor
Angpt1: Angiopoietin 1
BBB: Blood-brain barrier
Bcrp: Breast cancer resistance protein
CNS: Central nervous system
C1galt1: Core 1 synthase, glycoprotein-N-acetylgalactosamine 3-beta-galactosyltransferase 1
Cldn5: Claudin5
Col4a2: Collagen type IV α2
Cp: Ceruloplasmin
Cspg4: Chondroitin sulfate proteoglycan 4
Cxcr4: C-X-C motif chemokine receptor 4
Ddit4: DNA-damage-inducible transcript 4
Extl2: Exostosin like glycosyltransferase 2
Glut1: Glucose transporter 1
Gpc5: Glypican 5
GPCs: Glypicans
HIF: Hypoxia inducible factor
Hif1α: Hypoxia inducible factor 1α
Hs3st1: Heparan sulfate 3-O-sulfotransferase 1
JAK/STAT: Janus kinase/signal transducer and activator of the transcription
Lama5: Laminin alpha 5
LAT1: L-type amino acid transporter 1
Lrp1: Low-density lipoprotein receptor-related protein 1
Mmp9: Matrix metallopeptidase 9
mTOR: Mechanistic target of rapamycin kinase
NG2: Neuron-glial antigen 2
Nr3c1: Nuclear receptor subfamily 3 group C member 1
NVU: Neurovascular unit
PBS: Phosphate-buffered saline
Pdgfrb: Platelet derived growth factor receptor-β
PFC: Prefrontal cortex
P-gp: Permeability-glycoprotein
Pik3ca: PI3K catalytic subunit alpha
ROS: Reactive oxygen species
RT-qPCR: Real-time quantitative PCR
Sdc4: Syndecan 4
SDCs: Syndecans
Timp-3: Tissue inhibitor of metalloproteinase-3
Zfpm2: Zinc finger protein FOG family member 2
