# Supplementary material for: Sex- and age- differences in the expression of critical blood-brain barrier regulators: a physiological context
Source: Biol Sex Differ. 2025 Sep 2;16:67. doi: 10.1186/s13293-025-00751-2 (PMC12403491; doi:10.1186/s13293-025-00751-2)
Supplement: Supplementary file 1 — Supplementary Material 1. [file 13293_2025_751_MOESM1_ESM.docx]

**Supplementary Experimental Methods**

**Primer efficiency and specificity verification experiment.**

A template DNA sample of a given concentration was serially diluted (10 ×, 100 ×, and 1000 ×). qPCR amplification was performed using the same primer pair for all dilutions. The Ct value (y-axis) was plotted against the logarithm of the template concentration (log10 concentration, x-axis), and a standard curve was fitted. Primer efficiency was calculated by the slope of the standard curve with the formula: E = [-1 + 10^(-1/slope)] × 100%. An ideal primer efficiency should be between 95% and 105%.

Melting curve analysis was used to verify the specificity of the amplification product and eliminate interference from nonspecific products. A single main peak indicated the presence of a specific amplification product.

Figure legends

Figure S1. Melting Curve Analysis for each primer pair used in this study. (A) Adm. (B) Ager. (C) Angpt1. (D) B3gnt3. (E) BCRP. (F) C1gat1. (G) Cldn1. (H) Cldn5. (I) Col4a2. (J) Cp. (K) Cspg4. (L) Cxcr4. (M) Ddit4. (N) Extl2. (O) Galnt10. (P) Galnt2. (Q) Glut1. (R) Gpc5. (S) Hif1α. (T) Hs3st1. (U) Lama5. (V) LAT1. (W) Lrp1. (X) Mmp9. (Y) Nr3c1. (Z) Occludin. (AA) Pdgfrb. (AB) P-gp. (AC) Pik3cα. (AD) Sdc4. (AE) Socs3. (AF) Timp-3. (AG) Tjp1. (AH) Zfpm2. (AI) β-actin. (AJ) Cd31. (AK) Gfap. (AL) NeuN. (AM) Ttr.

Figure S2. Primer efficiency for each primer pair used in this study. (A) Adm. (B) Ager. (C) Angpt1. (D) B3gnt3. (E) BCRP. (F) C1gat1. (G) Cldn1. (H) Cldn5. (I) Col4a2. (J) Cp. (K) Cspg4. (L) Cxcr4. (M) Ddit4. (N) Extl2. (O) Galnt10. (P) Galnt2. (Q) Glut1. (R) Gpc5. (S) Hif1α. (T) Hs3st1. (U) Lama5. (V) LAT1. (W) Lrp1. (X) Mmp9. (Y) Nr3c1. (Z) Occludin. (AA) Pdgfrb. (AB) P-gp. (AC) Pik3cα. (AD) Sdc4. (AE) Socs3. (AF) Timp-3. (AG) Tjp1. (AH) Zfpm2. (AI) β-actin. (AJ) Cd31. (AK) Gfap. (AL) NeuN. (AM) Ttr.

Figure S3. Purity analysis of brain microvessel samples. The mRNA levels of Cd31, NeuN, Gfap and Ttr. Data are expressed as mean ± SEM. n = 12 per group. **** p < 0.0001, compared with the expression of Cd31.
