# Supplementary material for: Sex- and age- differences in the expression of critical blood-brain barrier regulators: a physiological context
Source: Biol Sex Differ. 2025 Sep 2;16:67. doi: 10.1186/s13293-025-00751-2 (PMC12403491; doi:10.1186/s13293-025-00751-2)
Supplement: Supplementary file 2 — Supplementary Material 2. [file 13293_2025_751_MOESM2_ESM.docx]

**Table S1 The efficiency of the primers**

| gene | Equation | R2 | E = ( -1+10^ (-1/slope)) *100% |
| --- | --- | --- | --- |
| Adm | Y=-3.235X+26.68 | 0.9830 | 103.75% |
| Ager | Y=-3.288X+25.96 | 0.9993 | 101.51% |
| Angpt1 | Y=-3.361X+24.74 | 0.9954 | 98.38% |
| B3gnt3 | Y=-3.210X+22.71 | 0.9954 | 104.88% |
| BCRP | Y=-3.272X+24.06 | 0.9986 | 102.12% |
| C1galt1 | Y=-3.221X+22.21 | 0.9822 | 104.41% |
| Cd31 | Y=-3.349X+24.99 | 0.9983 | 98.89% |
| Cldn1 | Y=-3.356X+26.02 | 0.9992 | 98.61% |
| Cldn5 | Y=-3.232X+21.81 | 0.9939 | 103.89% |
| Col4a2 | Y=-3.259X+24.44 | 0.9935 | 102.72% |
| Cp | Y=-3.424X+25.54 | 0.9929 | 95.93% |
| Cspg4 | Y=-3.330X+25.63 | 0.9999 | 99.66% |
| Cxcr4 | Y=-3.269X+26.27 | 0.9801 | 102.26% |
| Ddit4 | Y=-3.418X+24.81 | 0.9988 | 96.16% |
| Extl2 | Y=-3.216X+21.07 | 0.9966 | 104.64% |
| Galnt10 | Y=3.239X+27.74 | 0.9885 | 103.56% |
| Galnt2 | Y=-3.321X+23.16 | 0.9880 | 100.03% |
| Gfap | Y=-3.404X+23.27 | 0.9997 | 96.70% |
| Glut1 | Y=-3.280X+23.87 | 0.9995 | 101.79% |
| Gpc5 | Y=-3.436X+19.92 | 0.9808 | 95.48% |
| Hif1α | Y=-3.213X+19.79 | 0.9817 | 104.79% |
| Hs3st1 | Y=-3.266X+21.84 | 0.9991 | 102.35% |
| Lama5 | Y=-3.412X+23.79 | 0.9980 | 96.38% |
| LAT1 | Y=-3.221X+23.67 | 0.9953 | 104.41% |
| Lrp1 | Y=-3.222X+22.67 | 0.9990 | 104.36% |
| Mmp9 | Y=-3.411X+26.73 | 0.9988 | 96.38% |
| NeuN | Y=-3.407X+18.98 | 0.9934 | 96.56% |
| Nr3c1 | Y=-3.210X+22.52 | 0.9998 | 104.88% |
| Occludin | Y=-3.365X+25.41 | 0.9873 | 98.24% |
| Pdgfrb | Y=-3.220X+25.04 | 0.9981 | 104.41% |
| P-gp | Y=-3.237X+19.93 | 0.9989 | 103.70% |
| Pik3cα | Y=-3.218X+21.13 | 0.9987 | 104.55% |
| Sdc4 | Y=-3.233X+22.28 | 0.9998 | 103.84% |
| Socs3 | Y=-3.246X+21.68 | 0.9947 | 103.28% |
| Timp-3 | Y=-3.233X+22.21 | 0.9996 | 103.84% |
| Tjp1 | Y=3.290X+22.54 | 0.9997 | 101.33% |
| Ttr | Y=-3.327X+20.85 | 0.9974 | 99.80% |
| Zfpm2 | Y=-3.404X+21.64 | 0.9902 | 96.70% |
| β-actin | Y=-3.330X+19.82 | 0.9964 | 99.66% |
