# Supplementary figures and images for: Sex- and age- differences in the expression of critical blood-brain barrier regulators: a physiological context
Source: Biol Sex Differ. 2025 Sep 2;16:67. doi: 10.1186/s13293-025-00751-2 (PMC12403491; doi:10.1186/s13293-025-00751-2)

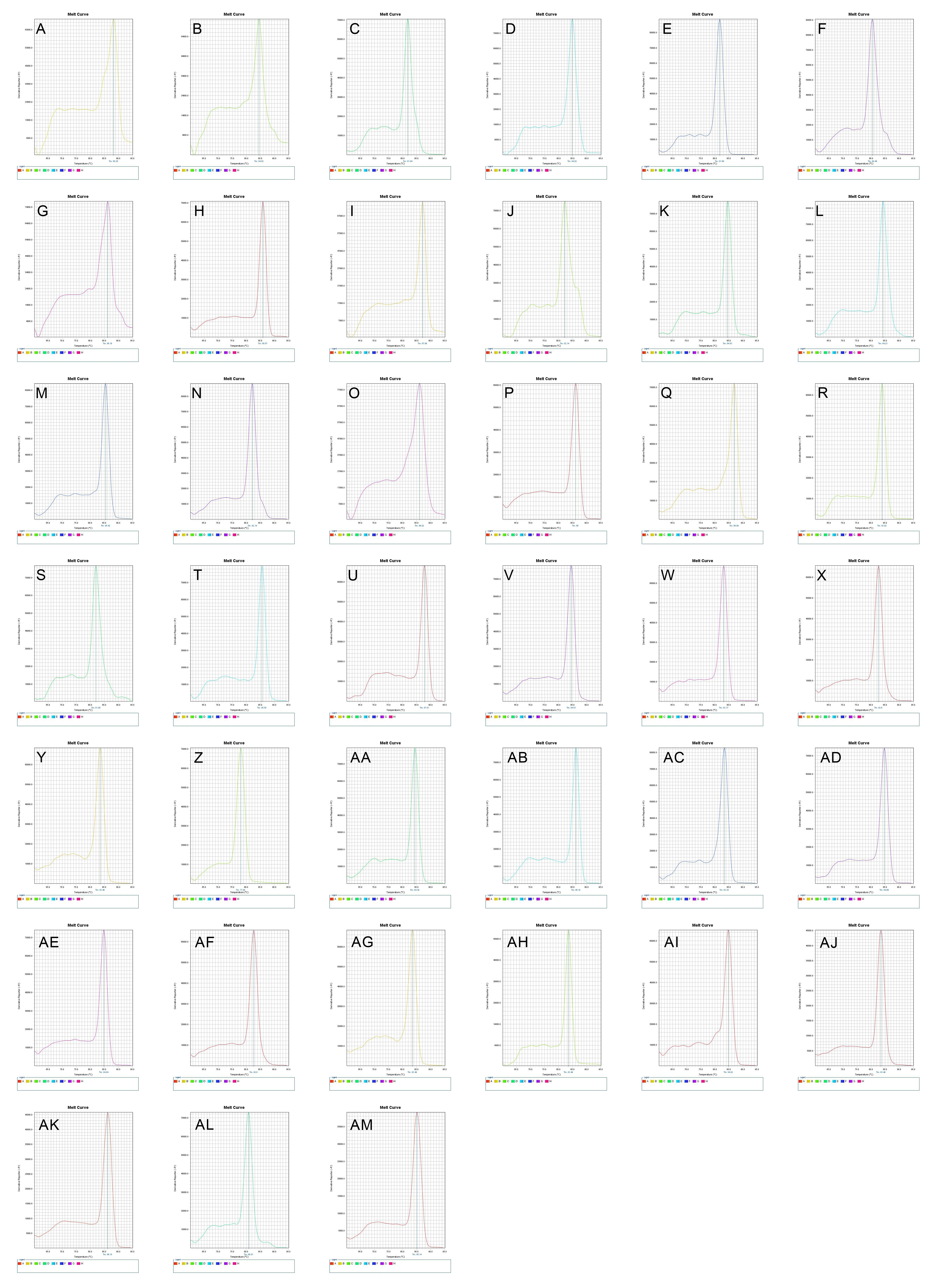

Supplement: Supplementary file 3 — Supplementary Material 3. [file 13293_2025_751_MOESM3_ESM.png]

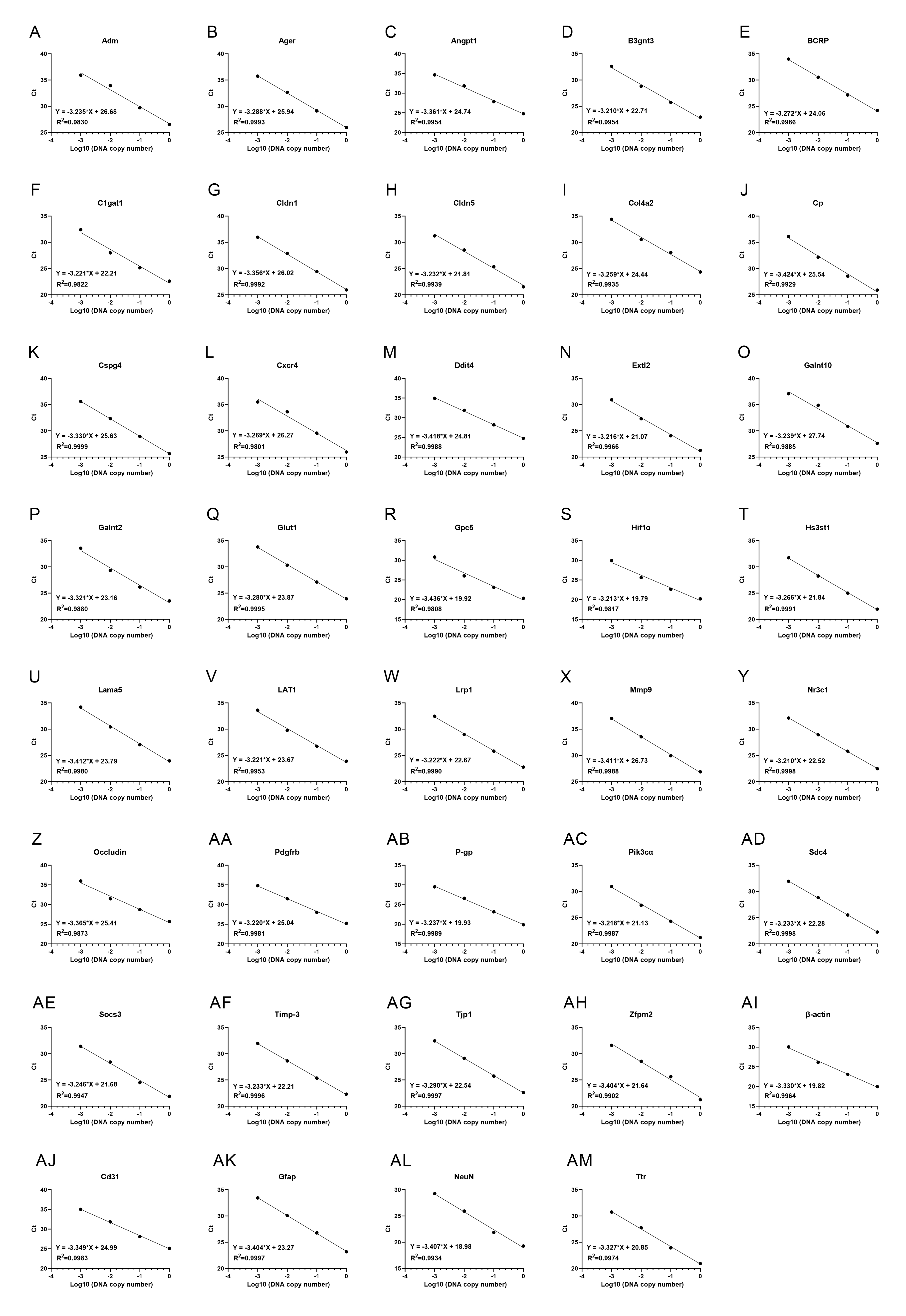

Supplement: Supplementary file 4 — Supplementary Material 4. [file 13293_2025_751_MOESM4_ESM.png]

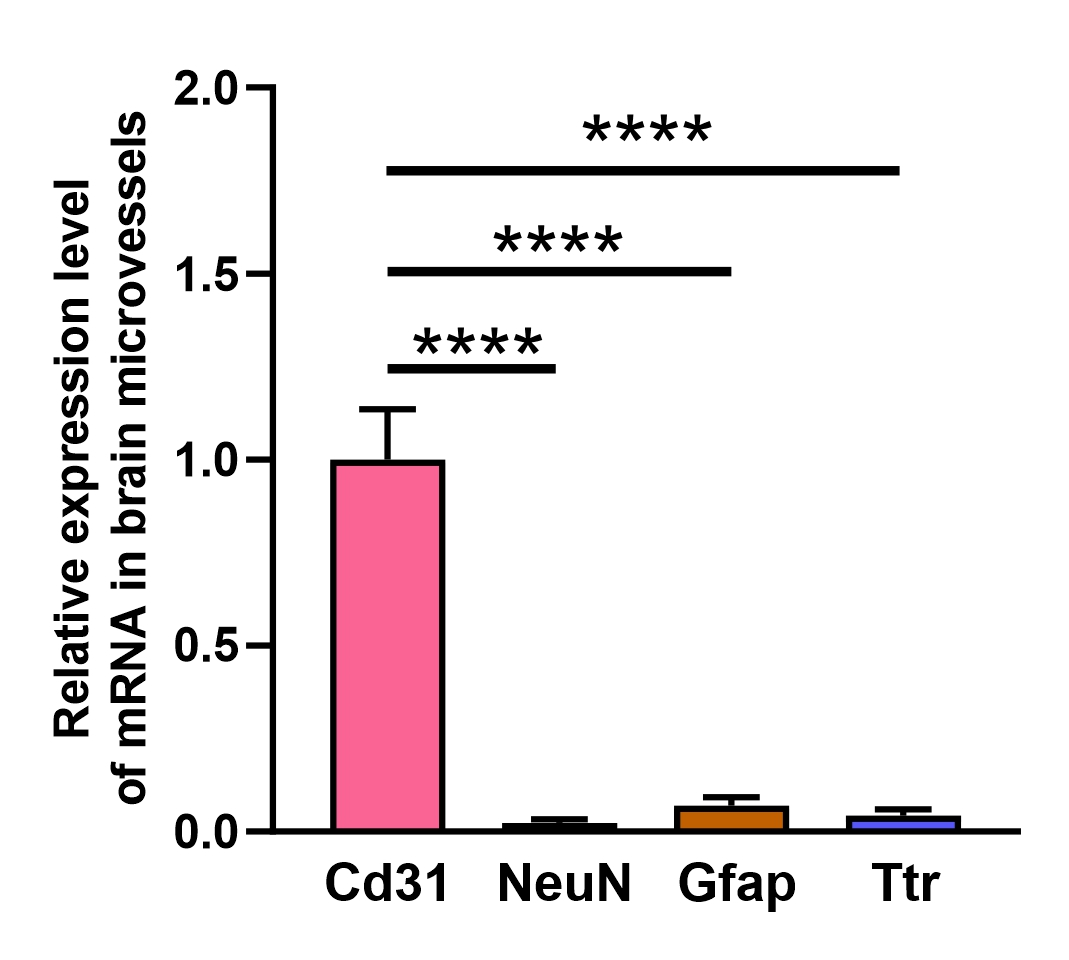

Supplement: Supplementary file 5 — Supplementary Material 5. [file 13293_2025_751_MOESM5_ESM.png]
